# Normal tissue complication probability model of temporal lobe injury following re-irradiation of CIRT for local recurrent nasopharyngeal carcinoma

**DOI:** 10.3389/fonc.2026.1853143

**Published:** 2026-06-26

**Authors:** Xiyin Guan, Jiayao Sun, Jiyou Peng, Xing Xing, Chaosu Hu

**Affiliations:** 1 Department of Radiation Oncology, Shanghai Proton and Heavy Ion Center, Fudan University Cancer Hospital, Shanghai, China; 2 Shanghai Key Laboratory of Radiation Oncology, Shanghai, China; 3 Shanghai Engineering Research Center of Proton and Heavy Ion Radiation Therapy, Shanghai, China; 4 Department of Medical Physics, Shanghai Proton and Heavy Ion Center, Fudan University Cancer Hospital, Shanghai, China; 5 Department of Radiation Oncology, Fudan University Shanghai Cancer Center, Shanghai, China; 6 Department of Oncology, Shanghai Medical College, Fudan University, Shanghai, China

**Keywords:** carbon ion radiotherapy, normal tissue complication probability model, recurrent nasopharyngeal carcinoma, re-irradiation, temporal lobe injury

## Abstract

**Purpose:**

Temporal lobe injury (TLI) is a significant complication after carbon-ion re-irradiation (reRT-CIRT) for locally recurrent nasopharyngeal carcinoma (rNPC). This study established normal tissue complication probability (NTCP) models for TLI based on (1) the second-course CIRT dose alone and (2) the cumulative dose of both radiotherapy courses, with the goal of defining clinically applicable, modality-specific dose constraints.

**Methods and materials:**

Eighty-nine rNPC patients who underwent reRT-CIRT between 2016 and 2020 were retrospectively analyzed. Deformable image registration transferred initial IMRT dose distributions onto CIRT planning scans, and voxel-wise cumulative equivalent doses in 2-Gy fractions (EQD_2_, α/β = 3 Gy) were calculated. Patients were stratified into training (n = 52) and validation (n = 37) cohorts. Multivariate logistic regression identified TLI predictors under two scenarios—second-course dose only versus cumulative two-course dose—and model performance was evaluated by ROC analysis.

**Results:**

TLI occurred in 27 patients (30.3%), predominantly unilateral (24/27); 14 were Grade 1 (asymptomatic, MRI-detected only), 7 Grade 2, 5 Grade 3, and 1 Grade 4. The second-course D_0.5_cc model [TD_5_ 34.27 Gy (RBE); TD_50_ 78.53 Gy (RBE); AUC 0.8450, 95% CI 0.7571–0.9329] and the cumulative D_5_cc model (TD_5_ 41.60 Gy; TD_50_ 124.31 Gy; AUC 0.8225, 95% CI 0.7228–0.9222) yielded equivalent and concordant risk stratification, with the inter-group dose difference primarily driven by the re-irradiation course. Compared with our previous photon-based reRT model, the CIRT-derived TD_50_ for D_0.5_cc and D_1_cc were ~20% higher (78.5 vs. 65.4 Gy; 76.8 vs. 62.9 Gy), indicating that photon-based constraints systematically overestimate TLI risk under CIRT.

**Conclusion:**

A CIRT-specific NTCP model based on the second-course D_0.5_cc achieves predictive performance equivalent to cumulative two-course dose summation, obviating prior-course dose reconstruction. This simplified, modality-specific tool offers practical guidance for individualized risk assessment and dose-constraint optimization in carbon-ion re-irradiation for rNPC.

## Highlights

- First NTCP model of temporal lobe injury (TLI) specifically derived for carbon-ion re-irradiation in recurrent nasopharyngeal carcinoma.
- A simplified second-course D_0.5_cc–based model achieves predictive performance equivalent to that of the cumulative two-course dose model (AUC 0.8450 vs. 0.8225), enabling reliable risk estimation when prior-course dosimetric data are unavailable.
- Voxel-wise EQD_2_ accumulation via deformable image registration provides a methodologically rigorous cross-validation of the simplified model.
- Clinically applicable CIRT-specific dose constraints proposed: D_0.5_cc TD_5_ = 34.27 Gy (RBE), TD_50_ = 78.53 Gy (RBE).
- CIRT-derived TD_50_ values are approximately 20% higher than photon-based constraints, indicating that photon-derived limits systematically overestimate TLI risk in the CIRT setting.
- Favorable CIRT toxicity profile: low rate of bilateral injury (3/27) and high proportion of asymptomatic, MRI-only TLI (14/27).

## 1 Introduction

Locally recurrent nasopharyngeal carcinoma (NPC) represents one of the primary modes of treatment failure following initial radiotherapy (1, 2). Carbon ion radiotherapy (CIRT) has shown dosimetric and clinical advantages over intensity-modulated radiotherapy (IMRT) in the re-irradiation of rNPC, offering improved dose conformity and potentially reduced toxicity (3, 4). However, temporal lobe injury (TLI) remains a significant complication after re-irradiation, particularly when the recurrent lesion is in close proximity to the temporal lobe (5). Even with the physical advantages of CIRT, the risk of TLI cannot be entirely eliminated.

Our group has previously established a normal tissue complication probability (NTCP) model for TLI after re-irradiation with IMRT (6). Nevertheless, there is a lack of data regarding dose-volume constraints for temporal lobes following CIRT re-irradiation. The biological effectiveness and dose calculation models for carbon ions-distinct from photon-based therapy-pose additional challenges for accurate dose accumulation and toxicity prediction. Therefore, this study aimed to develop an NTCP model for TLI in patients with rNPC undergoing CIRT re-irradiation, and to compare it with the existing model derived from IMRT re-irradiation.

## 2 Method and materials

### 2.1 Inclusion and exclusion criteria

The inclusion and exclusion criteria for the NTCP model of TLI following CIRT in this study align with those of the prior NTCP model study on TLI after IMRT re-irradiation (6).In brief, this study included patients with locally recurrent nasopharyngeal carcinoma (rNPC) who were pathologically or radiologically diagnosed, had no distant metastasis, demonstrated good performance status (KPS ≥70), and underwent MRI follow-up for at least 6 months after carbon-ion re-irradiation (CIRT). The distinct feature of this study is that all enrolled patients received CIRT as the re-irradiation modality. TLI was assessed via MRI with independent radiological review by two specialists; discrepancies were resolved by consensus, and residual or progressive disease was excluded from TLI site determination.

### 2.2 CT simulation and treatment planning​​

All initial-course intensity-modulated radiotherapy (IMRT) plans in this study were developed at the radiotherapy center of Fudan University Shanghai Cancer Center. CIRT was performed in Shanghai Proton and Heavy Ion Center. Patients were enrolled in the simulation, planning, and treatment workflow only after meeting the indications for CIRT and providing written informed consent for the CIRT treatment. A thermoplastic mask, heated to 60-82°C, was molded to conform to each patient’s head, neck, and shoulder contours for immobilization. The CT simulation scan extended from 1.5 cm above the cranial vertex to at least 2 cm below the clavicle, using a slice thickness of 1.5 mm. Following image acquisition, magnetic resonance imaging (MRI) was fused with the simulation CT to facilitate target delineation. Structures contoured included the gross tumor volume (GTV), clinical target volume (CTV), corresponding planning target volume (PTV), and organs at risk (OARs) relevant to nasopharyngeal carcinoma radiotherapy. The PTV was generated by adding a 3 to 5 mm margin around the CTV to account for setup and range uncertainties. Dose optimization prioritized sparing critical OARs-such as the optic nerves/chiasm, brainstem, spinal cord, and temporal lobes, particularly when they were adjacent to the target.

The prescribed dose was 3 Gy (RBE) per fraction for GTV, to a total dose of 60–69 Gy (RBE) in 20–23 fractions. The treatment plan was generated by Syngo TPS (V13B, Siemens, Mannheim, Germany) using three fields using the intensity modulated particle therapy (IMPT) technique. The gantry angle was fixed at 90°, and two lateral fields (left and right) as well as one vertex field were achieved by rotating the treatment couch. The RBE of CIRT was calculated based on the local effect model I (LEM). Planning objectives aimed to cover ≥95% of the target volume with 100% of the prescription dose, and ≥95% of the PTV with 90% of the prescription dose, while ensuring that dose constraints to critical organs were respected. Treatment was delivered once daily, five days per week. Prior to irradiation, setup verification was performed using a pair of orthogonal X-ray portal images, with tolerance limits of <1 mm for axial displacement and <1°for rotation.

### 2.3 EQD_2_ conversion and dose volume histogram data calculation

In this study, the grading of TLI was based on the CTCAE version 4.0 criteria. The methodological approach aligns with our prior IMRT-based NTCP study (6). Dose accumulation and volumetric analysis were performed using MIM software (v6.5.9) to integrate initial photon radiotherapy and carbon ion re-irradiation plans. Bilateral temporal lobes were re-delineated on re-irradiation CT scans with left/right structures defined separately. For confirmed TLI cases, contrast-enhanced MRI was fused to CT; injury volume was defined strictly as the necrotic core with ring enhancement on T1-weighted images, excluding surrounding edema. Overlapping regions between target volumes and temporal lobes were incorporated into the target for dosimetric accuracy.

Dose mapping was performed using deformable image registration within the MIM platform (v6.5.9, Cleveland, OH, USA), aligning the initial IMRT plan with the CIRT re-irradiation CT dataset. Dose accumulation accounted for differing fractionation schedules by converting physical doses to EQD_2_via the linear-quadratic model (α/β = 3 Gy for temporal lobe tissue), following established radiobiological principles (7). All dose calculations were executed in Python (v3.9.6), with cumulative EQD_2_ values derived at 0.1 Gy resolution. Key DVH metrics—including Dmax, D0.5–5.0cc (dose to 0.5–5.0 cc volumes in 0.5 cc increments), and dose contributions from both treatment courses—were extracted for bilateral temporal lobes and TLI subregions.


$$\text{EQD}2=\text{D}x\;\frac{\frac{\alpha }{\beta }+{\text{d}}_{x}}{\frac{\alpha }{\beta }+{\text{D}}_{2}}$$

### 2.4 Construction and validation of the NTCP model

Eligible patients were allocated to training and validation cohorts. The inter-treatment interval was defined as the duration between completion of initial radiotherapy and initiation of re-irradiation. TLI occurrence was assessed from the conclusion of re-irradiation, while patient age represented the age at recurrent nasopharyngeal carcinoma diagnosis. ROC curve analysis identified the DVH parameter with maximal discriminatory power for TLI prediction. Univariate and multivariate binary logistic regression evaluated the influence of DVH metrics and clinical factors on TLI risk, with stepwise regression applied to determine optimal dose-volume thresholds.

NTCP model was established based on the following formula, with the bilateral temporal lobes of each patient separately included in the analysis:


$$\text{NTCP}=\frac{1}{1+e^{-(a+\sum_{i=1}^{m}b_{i}x_{i})}}$$

Where x1, x2,…, xm represent predictive variables (dosimetric and/or clinical factors), and a, b1, b2,…, bm denote the regression coefficients derived from the model. Where Dx is the total physical dose delivered in fraction size dx and D_2_ = 2 Gy denotes the reference fraction size.

Model robustness was assessed by deriving tolerance doses (TD_5_ and TD_50_) with 95% confidence intervals, alongside ROC curve analysis to determine AUC and optimal diagnostic thresholds. Dose-response relationships between TLI risk and dosimetric parameters were visualized via nonlinear logistic regression. The model was internally optimized by iterative parameter fitting under maximum-likelihood estimation, and externally evaluated on the validation cohort using ROC-derived sensitivity and specificity. All analyses utilized SPSS v26.0 (IBM Corp., Chicago, IL, USA), with two-tailed p ≤ 0.05 deemed statistically significant.

Subsequently, multivariate logistic regression was performed under two analytical scenarios designed to reflect clinical variability in the availability of prior-course dosimetric data: (1) second-course dose only—applicable both when the initial radiotherapy plan is unavailable and when the two plans are available but voxel-wise dose accumulation is not feasible; and (2) cumulative two-course dose—applied when both plans are available and full EQD_2_-based dose accumulation can be performed. Comparing the two scenarios allowed us to evaluate whether the simplified second-course-only model could provide predictive performance equivalent to the more complex cumulative-dose model.

## 3 Results

A total of 89 patients with recurrent nasopharyngeal carcinoma who received carbon-ion re-irradiation between January 2016 and December 2020 were included in this study, the median follow-up time was 41.3 months (range, 8.2-112.5 months). All patients had complete clinical records, and complete physical radiotherapy plans for both courses. To ensure comparability with our previous study, which utilized a training set of 52 patients, the cohort in this study was similarly allocated—with 52 patients in the training set and 37 in the validation set. **Table 1** summarizes the clinical characteristics of the two cohorts, showing no statistically significant differences between the groups. TLIs occurred in 27 patients, yielding an incidence of 30.3%. Among these, 3 patients had bilateral TLI, while the remaining had unilateral involvement. The enhanced and necrotic regions on MRI were delineated, resulting in a median TLI volume of 4.7 cc (range: 0.5–34.8 cc). The median time to TLI development was 12.0 months (range: 3.2–28.2 months). Among the 27 patients with TLI, 14 were Grade 1 (asymptomatic, identified only by MRI findings), 7 were Grade 2, 5 were Grade 3, and 1 was Grade 4. There were no Grade 5 (fatal) cases. Of the symptomatic patients, the most common presentations were headache and dizziness (10 patients), followed by memory decline (3 patients). No patient exhibited personality changes.

**Table 1:** Clinical characteristics of 89 patients with recurrent nasopharyngeal carcinoma.

Clinical characteristics		Training set No. (%)	Validation set No. (%)	P value
Gender	Male	38 (73.1)	25 (67.6)	0.573
	Female	14 (26.9)	12 (32.4)	
	Median age (y) (range)	45 (17-71)	48 (23-78)	0.746
T stage of primary tumor	1-2	21 (40.4)	15 (40.6)	0.746
	3	13 (25.0)	11 (29.7)	
	4	18 (34.6)	11 (29.7)	
N stage of primary tumor	0-1	32 (61.5)	14 (53.8)	0.261
	2-3	20 (38.5)	23 (46.1)	
RT dose of primary tumor	66	25 (48.1)	18 (48.6)	0.958
	70.4	27 (51.9)	19 (51.4)	
Interval (months) between 2 RT (range)		31.5 (13.1-391)	29.3 (11.0-90.3)	0.762
rT stage	0-2	24 (46.1)	11 (29.7)	0.610
	3	11 (21.2)	11 (29.7)	
	4	17 (32.7)	15 (40.6)	
rN stage	0	39 (75.0)	28 (75.7)	0.470
	1	13 (25.0)	9 (24.3)	
Dose of carbon ion radiotherapy	60	8 (15.4)	7 (18.9)	0.318
	63	20 (38.5)	19 (51.4)	
	66	18 (34.6)	10 (27.0)	
	69	6 (11.5)	1 (2.7)	
Induction chemo	Yes	21 (40.4)	19 (51.4)	0.305
	No	31 (59.6)	18 (48.6)	
Concurrent chemo	Yes	15 (28.8)	12 (32.4)	0.717
	No	37 (71.2)	25 (67.6)	
Temporal lobe injury	Yes	17 (32.7)	10 (27.0)	0.567
	No	35 (67.3)	27 (73.0)	

**Figure 1:**
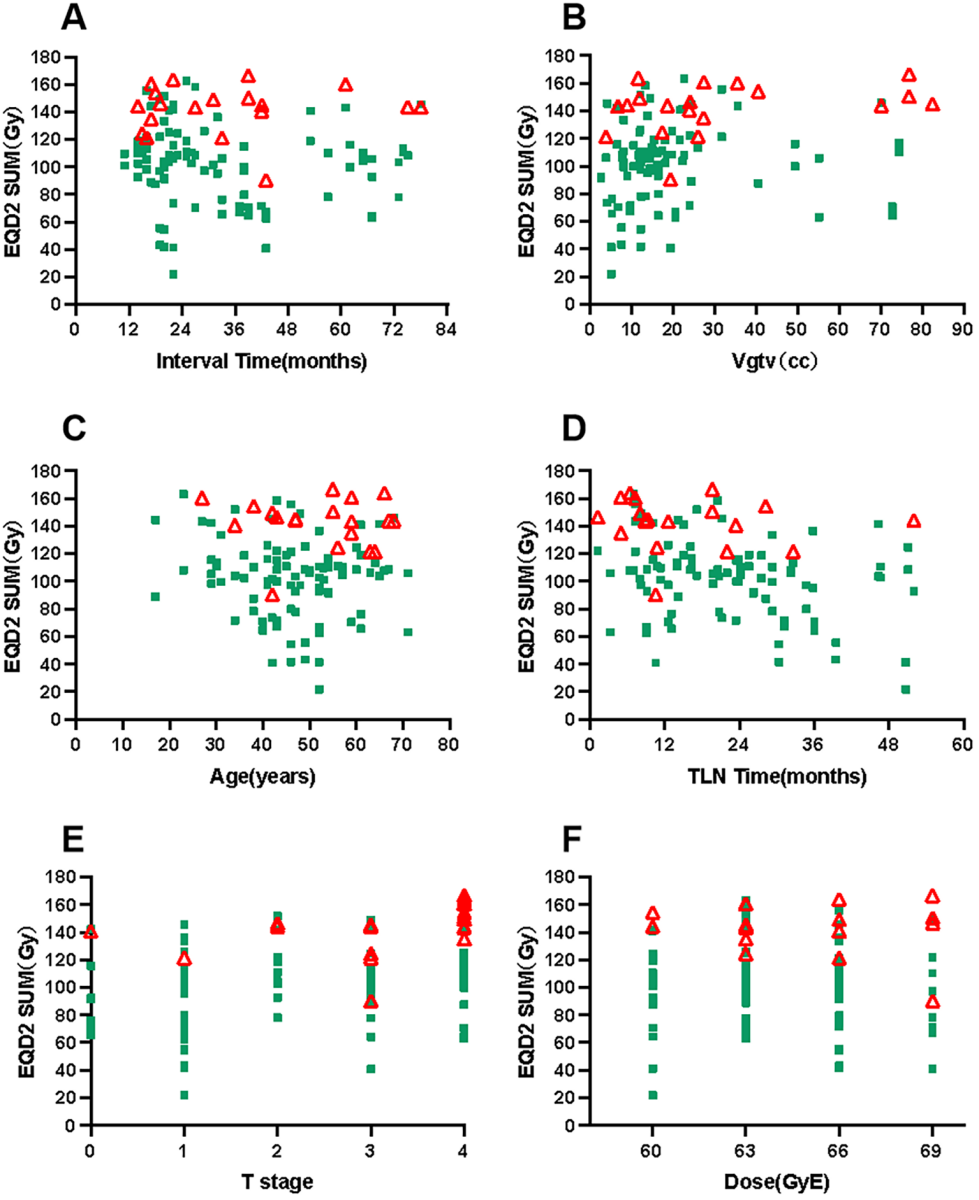
Scatter plots illustrating the association between potential influencing factors and the occurrence of temporal lobe injury (TLI) following carbon-ion re-irradiation for recurrent nasopharyngeal carcinoma. The y-axis represents the cumulative D_0.5_ cc, expressed as the equivalent dose in 2-Gy fractions (EQD_2_; α/β = 3 Gy), summed from the two radiotherapy courses. Red triangles denote patients who developed TLI, whereas green squares denote patients without TLI. **(A)** Interval between the two radiotherapy courses (months); **(B)** Gross tumor volume (GTV) at recurrence (cm³); **(C)** Patient age at recurrence (years); **(D)** Time to TLI onset after carbon-ion radiotherapy (CIRT) (months); **(E)** T-stage at recurrence; **(F)** Re-irradiation prescription dose [Gy (RBE)].

The relationship between various potential clinical factors associated with TLI and the occurrence of TLI is illustrated in the scatter plots in **Figure 1**. No significant correlation was observed between the interval between the two courses of radiotherapy and the development of TLI. A larger gross tumor volume (GTV) was associated with a higher proportion of TLI. Similarly, since larger tumor volume often correlates with more advanced disease stage, patients with higher T-stage also showed an increased probability of TLI. Older age tended to be associated with a higher proportion of TLI, and a higher prescription dose was also related to an increased likelihood of TLI. TLI occurred more frequently among older patients, and most cases developed within approximately one year after re-irradiation. However, none of these factors reached statistical significance.

The results of the TLI NTCP model following re-irradiation with CIRT are presented in **Table 2**. When the initial and re-irradiation plans were not summed, and the doses from the two courses were calculated separately, the results were consistent with those considering only the volumetric dosimetric parameters of the re-irradiation. However, after cumulative dose summation, in addition to the corresponding DVH parameters, the GTV volume and the prescription dose of CIRT should also be considered. As shown in **Table 3**, no significant differences were observed in the first-course IMRT dose parameters between patients who developed TLI and those who did not. However, the temporal lobe dose delivered in the re-irradiation of CIRT was significantly higher in patients with injury than in those without. When the EQD_2_ doses from both courses were summed, the cumulative dose to the temporal lobes for the injured patients was significantly higher than that in uninjured patients. Therefore, we conclude that the difference in the cumulative dose between the two groups was primarily attributable to the difference in the second-course carbon-ion dose.

**Table 2:** Factors associated with temporal lobe injury (TLI) in the binary multivariate logistic regression model.

	Pvalue	Wald	95% CI	Regression equation	Nagelkerke R²
Scenario 1: Second-course dose only					
2^nd^ D_0.5_ cc	<0.001	18.835	1.055-1.153	S=0.098*2^nd^D0.5-7.474	0.474
Scenario 2: Cumulative dose after EQD_2_ summation					
D_5_cc	<0.001	16.405	1.024-1.067	S=0.058*D5+ 0.030*2^nd^VGTV +0.477 *2^nd^ Dose -5.251	0.330
2^nd^ VGTV	0.045	4.014			
2^nd^ Prescription Dose	0.008	6.983			

2nd D_0.5_cc, maximum EQD_2_ dose delivered to 0.5 cc of the temporal lobe during the second (re-irradiation) CIRT course [Gy (RBE)]; Sum D_5_cc, cumulative EQD_2_ dose received by 5 cc of the temporal lobe after voxel-wise summation of the initial IMRT and re-irradiation CIRT courses [Gy (RBE)]; 2nd V_GTV, gross tumor volume of the recurrent lesion at re-irradiation (cm³); 2nd Prescription Dose, prescribed dose of the CIRT re-irradiation course [Gy (RBE)]; CI, confidence interval.

Predictors of TLI were identified by binary multivariate logistic regression under two analytical scenarios: (1) using the second-course CIRT dose alone—applicable both when the initial plan is unavailable and when both plans are available but voxel-wise dose accumulation is not feasible; and (2) using the cumulative two-course dose after voxel-wise EQD_2_ summation. The probability of TLI is calculated as P = 1/(1 + e^−S), where S denotes the logit value from the regression equation. All doses are expressed in Gy (RBE) for CIRT and as EQD_2_ (α/β = 3 Gy) for cumulative dose. P < 0.05 was considered statistically significant.

**Table 3:** Comparison of dose-volume parameters between injured and non-injured temporal lobes within patients (Training set, N = 52 patients, 104 temporal lobes analyzed).

Variable	T-value	P-value	Mean difference	Standard deviation	Lower limit	Upper limit
IMRT-D_3_	1.381	0.170	5.459	3.954	-2.377	13.294
IMRT-D_2.5_	1.276	0.204	5.126	4.016	-2.832	13.084
IMRT-D_2_	1.260	0.210	5.138	4.078	-2.943	13.219
IMRT-D_1.5_	1.209	0.229	5.013	4.147	-3.206	13.232
IMRT-D_1_	1.142	0.256	4.824	4.222	-3.544	13.192
IMRT-D_0.5_	1.175	0.243	5.083	4.326	-3.490	13.656
IMRT-Dmax	1.019	0.310	4.692	4.602	-4.429	13.813
CIRT-D_3_	7.716	<0.01	37.783	4.897	28.078	47.487
CIRT-D_2.5_	7.659	<0.01	37.987	4.960	28.157	47.816
CIRT-D_2_	7.620	<0.01	38.139	5.005	28.221	48.058
CIRT-D_1.5_	7.540	<0.01	37.872	5.022	27.918	47.825
CIRT-D_1_	7.422	<0.01	37.431	5.043	27.436	47.426
CIRT-D_0.5_	6.993	<0.01	35.799	5.119	25.654	45.944
CIRT-Dmax	5.529	<0.01	29.987	5.423	19.240	40.735
SUM-D_3_	6.548	<0.01	43.242	6.604	30.154	56.330
SUM-D_2.5_	6.466	<0.01	43.113	6.668	29.899	56.327
SUM-D_2_	7.539	<0.01	43.277	5.740	31.610	54.945
SUM-D_1.5_	6.376	<0.01	42.884	6.726	29.555	56.214
SUM-D_1_	6.270	<0.01	42.255	6.739	28.900	55.609
SUM-D_0.5_	6.042	<0.01	40.882	6.766	27.473	54.291
SUM-Dmax	4.934	<0.01	34.679	7.029	20.749	48.609

Dmax, maximum dose; D0.5–D3, dose to 0.5–3 cc of the temporal lobe [Gy (RBE)]; IMRT, intensity-modulated radiotherapy; CIRT, carbon ion radiotherapy; SUM, cumulative dose of IMRT plus CIRT.

Comparisons were performed using paired Student’s t-test between the injured and non-injured temporal lobes within the same patient. “Upper limit” and “Lower limit” refer to the 95% confidence interval of the mean difference. Doses are expressed in Gy (RBE) for carbon ion plans and Gy for photon plans. P < 0.05 was considered statistically significant.

**Figure 2:**
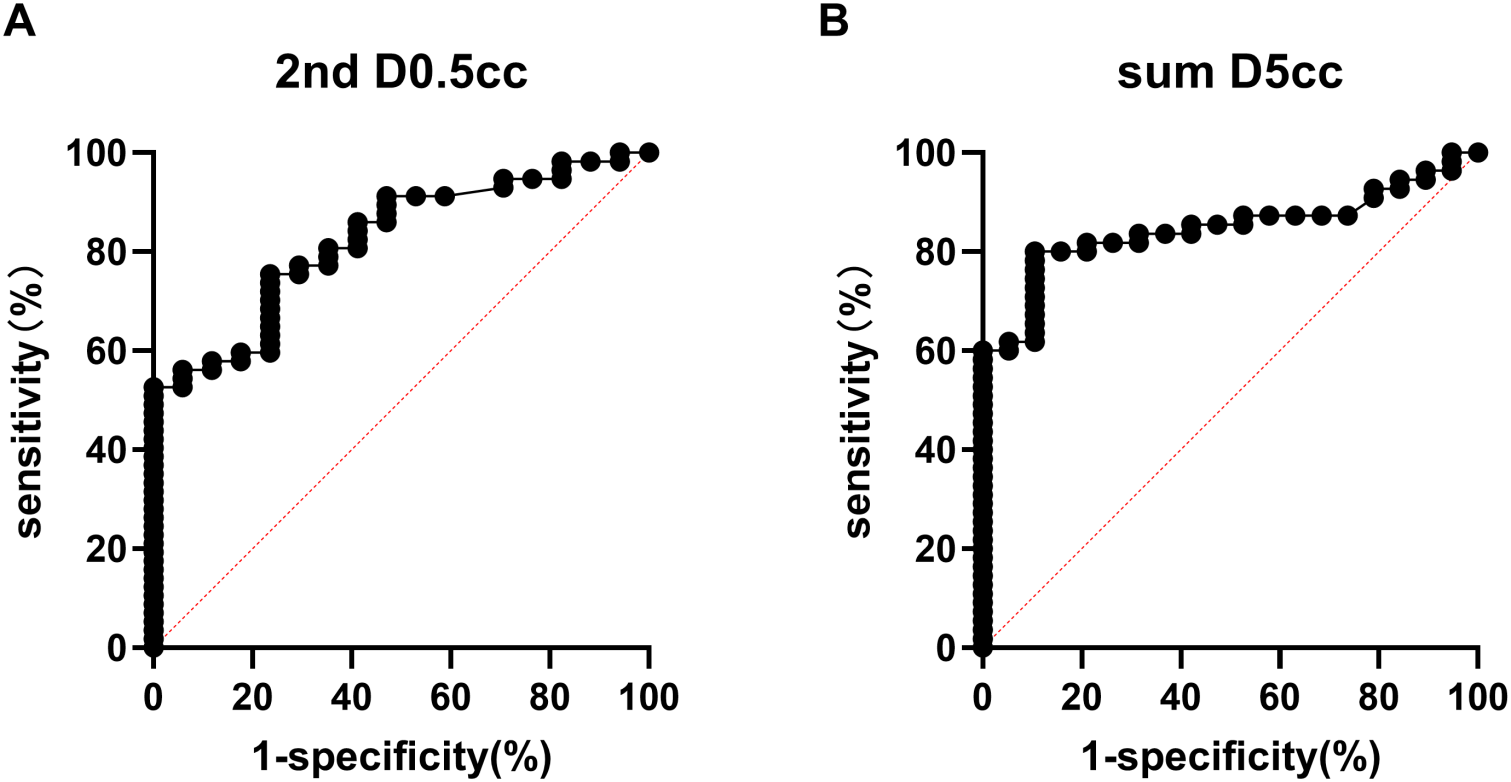
Dose–response curves derived from the normal tissue complication probability (NTCP) model for temporal lobe injury (TLI) at selected dose–volume points. **(A)** Predicted probability of TLI as a function of D_0.5_ cc from the carbon-ion re-irradiation course alone. **(B)** Predicted probability of TLI as a function of cumulative D_5_cc, calculated as the voxel-wise sum of the equivalent dose in 2-Gy fractions (EQD_2_; α/β = 3 Gy) from the initial intensity-modulated radiotherapy (IMRT) and the carbon-ion re-irradiation courses. Doses are expressed in Gy (RBE).

Based on the NTCP models, the EQD_2_dose corresponding to a 5% probability of injury for the second-course D_0.5_ cc was calculated to be 34.27 Gy (RBE) (15.57–57.71 Gy (RBE)), and the dose for a 50% injury probability was 78.53 Gy (RBE) [54.33–94.34 Gy (RBE)]. For the cumulative dose D_5_cc, the EQD_2_ dose for a 5% injury probability was 41.60 Gy (22.23–60.69 Gy), and for a 50% injury probability, it was 124.31 Gy (88.19–146.57 Gy). The fitted curves are shown in **Figure 2**.

As shown in **Figure 2**, the NTCP values corresponding to volumetric dosimetric parameters for patients in the validation cohort were calculated based on the NTCP model. These values were then evaluated against the actual occurrence of TLI using ROC curve analysis. When considering only the dose from the re-irradiation of radiotherapy, the area under the ROC curve (AUC) for the 2nd D_0.5_ cc was 0.8450 (95% CI: 0.7571–0.9329), with *p* < 0.0001.

**Figure 3:**
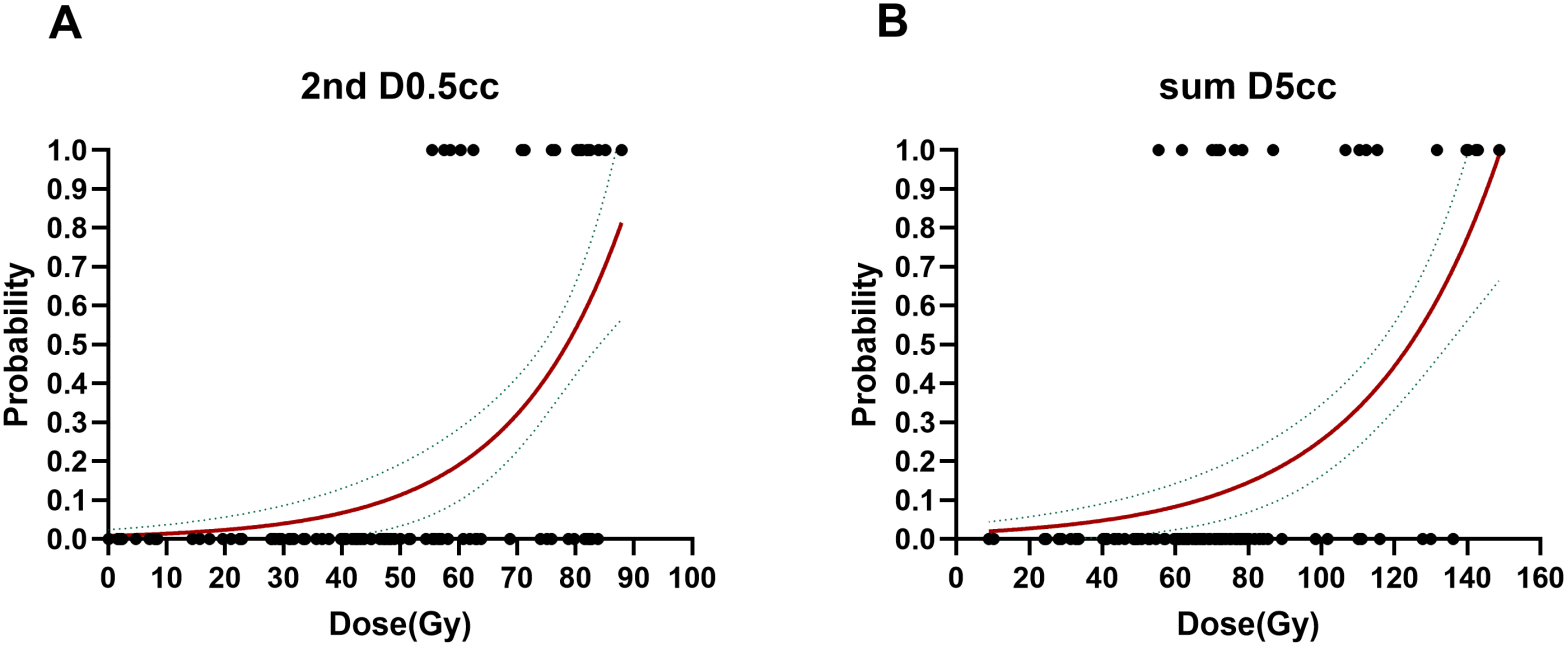
Receiver operating characteristic (ROC) curves of the NTCP model for predicting temporal lobe injury (TLI) in the validation cohort. **(A)** Based on D_0.5_cc from the carbon-ion re-irradiation course (AUC = 0.8450; 95% CI, 0.7571–0.9329; P < 0.0001). **(B)** Based on cumulative D_5_cc derived from voxel-wise EQD_2_summation (α/β = 3 Gy) of the initial IMRT and carbon-ion re-irradiation courses (AUC = 0.8225; 95% CI, 0.7228–0.9222; P < 0.0001). Doses are expressed in Gy (RBE). AUC, area under the curve; CI, confidence interval.

When the EQD_2_ doses from both the initial and re-irradiation were summed, the AUC for the sum D_5_cc was 0.8225 (95% CI: 0.7228–0.9222), also with *p* < 0.0001. The ROC curves were shown in **Figure 3**.

## 4 Discussion

The interval to the development of TLI following re-irradiation for rNPC is significantly shorter than that observed after initial radiotherapy. As a late-responding tissue, the temporal lobe typically manifests injury no sooner than three years after initial radiotherapy. Previous studies have reported a median time to TLI of 27–34 months following initial IMRT for NPC (8, 9). This latency period is substantially shortened after re-irradiation. Studies by Liu and Han et al. (5, 10) demonstrated a median time to TLI of 15 months after re-irradiation. Consistent with these findings, the present study observed a median time to TLI of 12 months, which aligns well with previously published data. In this study, a minimum follow-up period of six months post-re-irradiation was set for inclusion. We acknowledge the theoretical concern that this duration may be insufficient to capture all potential late-onset TLI. However, this design reflects the challenging clinical reality of the patient population. Outcomes following re-irradiation for recurrent nasopharyngeal carcinoma are often poor. Unpublished data from our center indicates a 2-year overall survival rate of less than 75% after carbon-ion re-irradiation. The patients with follow-up shorter than one year were predominantly those with rapid disease progression or who died, events that preclude the future development of TLI. Including these patients aligns with the actual clinical course and survival patterns of this cohort.

CIRT has demonstrated a potential to reduce the risk of TLI in patients with rNPC. While the reported incidence of TLI following primary IMRT for NPC is generally below 10% (11), re-irradiation for rNPC further elevates this risk. A study by Liu et al. (5) involving 227 patients reported TLI in 71 cases (31.3%), with nearly half (49.3%) presenting bilateral involvement. In the present study, TLI was observed in 27 patients (30.3%), only three of whom had bilateral injuries. Notably, 14 cases were asymptomatic, with injuries detected only on imaging. These subclinical injuries were typically small in volume and had negligible impact on patients’ quality of life. This favorable profile may be attributed to the superior dose distribution of CIRT, which achieves significant sparing of the contralateral temporal lobe compared to IMRT, where bilateral temporal lobes often receive considerable dose. For patients with locally rNPC, particularly those with T3–T4 disease, high-dose exposure to the temporal lobes is often unavoidable due to anatomical proximity between the target volume and temporal lobe tissue. In such cases, CIRT offers a critical advantage by maximizing target coverage while minimizing the volume and severity of TLI. In conclusion, carbon-ion re-irradiation for rNPC not only reduces the incidence of bilateral TLI but also mitigates the clinical impact of unilateral injury, thereby better preserving patients’ quality of life.

A central finding of this study is that the NTCP model derived solely from the second-course CIRT dose achieves predictive performance equivalent to that derived from the cumulative dose of both treatment courses, and yields concordant risk-stratification conclusions. Specifically, the second-course D_0.5_cc model achieved an AUC of 0.8450 (95% CI: 0.7571–0.9329) in the validation cohort, comparable to—and numerically slightly higher than—the cumulative D_5_cc model (AUC = 0.8225, 95% CI: 0.7228–0.9222). This concordance is not coincidental but reflects the underlying dosimetric reality of contemporary rNPC management. Over the past decade, the standardization of IMRT for NPC has led to increased consistency in target volume delineation, particularly for CTVs, resulting in relatively uniform doses to the temporal lobes during primary treatment (12). For patients with recurrent nasopharyngeal carcinoma, radiation is delivered only to the tumor target volume without prophylactic irradiation of anatomical regional structures. As demonstrated in **Table 3**, no significant differences were observed in the first-course IMRT dose parameters between patients who developed TLI and those who did not, whereas the re-irradiation CIRT dose to the temporal lobe was significantly higher in injured patients. Consequently, the inter-group difference in cumulative temporal lobe dose was almost entirely driven by the re-irradiation course, with negligible contribution from the initial treatment.

From a clinical workflow perspective, this finding has substantial practical implications. Accurate dose accumulation requires retrieval of the original IMRT plan, deformable image registration between the two CT datasets, voxel-wise EQD_2_ conversion, and quality assurance of the registration accuracy—steps that are time-consuming, software-dependent, and frequently infeasible when initial-course dosimetric data are missing, archived in incompatible formats, or originate from external institutions. By contrast, the second-course D_0.5_cc can be extracted directly from the CIRT planning system at the time of re-irradiation planning, requiring no additional infrastructure or computational workflow. Therefore, for rNPC patients in whom detailed dosimetric data from the first course are unavailable—or when rapid clinical decision-making is required—the second-course D_0.5_cc-based model proposed in this study provides a reliable and readily deployable surrogate for cumulative-dose-based risk estimation. Based on the NTCP model derived solely from the second-course dose, the EQD_2_ values corresponding to a 5% complication probability (TD_5_) and a 50% complication probability (TD_50_) for D_0.5_cc were 34.27 Gy (15.57–57.71 Gy) and 78.53 Gy (54.33–94.34 Gy), respectively, which can serve as direct reference constraints in routine CIRT treatment planning.

The cumulative-dose NTCP model in this study serves primarily as a methodological validation of the second-course-only model, rather than as the primary clinical decision tool. Accurately quantifying the cumulative radiation dose to the temporal lobe following two courses of radiotherapy has been a persistent challenge due to the complexities of dose superposition. To address this, the present study implemented a rigorous methodology: the dose distribution from the initial treatment plan was mapped onto the re-irradiation plan via deformable image registration, and the dose to each voxel within the temporal lobe was converted to EQD_2_ before summation. This approach effectively resolved the inaccuracies inherent in simple physical dose addition. Based on the derived NTCP model, the TD_5_ for the cumulative D_5_cc was calculated to be 41.60 Gy (95% CI: 22.23–60.69 Gy), and the TD_50_ was 124.31 Gy (95% CI: 88.19–146.57 Gy). In a previous study by Liu et al. (5), which analyzed 227 NPC patients receiving 2D-conventional radiotherapy followed by IMRT re-irradiation, the sum of the maximum TL doses from both courses was identified as an independent predictor of injury. However, that study relied on estimated doses from the first course and the accuracy of direct summation without EQD_2_ conversion remains questionable. A study on brain re-irradiation suggested that a cumulative EQD_2_ sum of less than 96 Gy is considered safe for two courses of 2D radiotherapy, with a complication probability of 0–3% for a cumulative EQD_2_ below 101 Gy (13). The methodology employed in this study is more comprehensive than previous literature, effectively resolving the challenge of dose superposition for normal tissue injury after two courses of radiotherapy. Importantly, the convergence between the cumulative-dose model and the second-course-only model—both yielding strong and statistically equivalent discrimination—provides internal cross-validation that strengthens confidence in the second-course D_0.5_cc as a standalone clinical predictor.

Carbon ions are high-LET radiation, whose RBE and EQD_2_ conversion require further validation, while also exhibiting differences in fraction dose and radiobiological effects compared to photon radiotherapy (14, 15). Therefore, dose summation based on EQD_2_ between the two treatment modalities remains debatable. In this study, relying on the RBE-weighted values from the LEM model and then applying LQ-model-based EQD_2_ conversion may lead to double-counting or produce mixed parameters with unclear biological-effect relationships. For instance, the application of the LQ model in particle therapy is controversial, yet no more suitable alternative currently exists for comparing photon and CIRT, and it remains widely used for dose conversion in particle centers worldwide (16, 17). Furthermore, in CIRT, both the LEM and the Microdosimetric Kinetic Model (MKM) are used; under the same physical dose, the bioequivalent doses calculated by these two models based on RBE differ. The LEM employed in this study may potentially overestimate the equivalent biological dose (14).

A direct comparison with our previously established photon-based reRT NTCP model (6) further underscores the necessity of modality-specific dose constraints. When only re-irradiation parameters were considered, the strongest predictive parameter (EQD_2_) for TLI was the second-course D_1_cc in IMRT re-irradiation, whereas in CIRT re-irradiation it was the second-course D_0.5_cc—suggesting that CIRT-induced injury is driven by even smaller high-dose volumes, consistent with its sharper dose gradient. The TD_50_ values further differed substantially between modalities: for CIRT, D_0.5_cc and D_1_cc were 78.5 Gy (RBE) and 76.8 Gy (RBE), respectively, while for photons, the corresponding values were 65.4 Gy and 62.9 Gy, yielding ratios of 120% and 122%. In other words, applying photon-derived TLI dose constraints to CIRT re-irradiation would systematically overestimate the complication risk by approximately 20%, potentially resulting in unwarranted dose de-escalation and compromised tumor control. This finding emphasizes that NTCP models cannot be directly transferred between radiation modalities, and that CIRT-specific dose constraints—such as those proposed in the present study—are essential for evidence-based treatment planning in rNPC re-irradiation.

Several limitations should be acknowledged. First, certain methodological choices were made to preserve direct comparability with our previously published photon-based reRT NTCP model (6), which is central to the cross-modality comparison pursued in this work. Specifically, bilateral temporal lobes were entered separately into the logistic regression, and the 52/37 training–validation split mirrored the prior photon cohort. We recognize that treating two lobes from the same patient as independent observations introduces within-patient correlation that is not fully accommodated by standard logistic regression, and that this pragmatic split was not based on a prospectively defined power calculation. Future analyses incorporating generalized estimating equations or mixed-effects models, together with bootstrap-based internal validation, would further refine model precision. Second, TLI was analyzed as a binary endpoint rather than within a time-to-event framework. A minimum follow-up of 6 months with complete records was required for inclusion; extending this threshold to 12 or 18 months would have substantially reduced the analyzable cohort, given the relative scarcity of recurrent NPC patients receiving CIRT. A time-to-event analysis in larger multicenter cohorts is therefore planned. Third, although deformable image registration (DIR) and EQD_2_ conversion were performed with careful visual quality assurance, the spatial uncertainty of DIR and the potential biological ambiguity arising from sequential LEM-based RBE weighting and LQ-based EQD_2_ conversion were not exhaustively quantified. Notably, the proposed second-course-only model is inherently less susceptible to these uncertainties, supporting its preferential use in routine practice. Finally, the model was internally validated within a single-center cohort, and external validation in independent multicenter datasets is warranted.

## Data Availability

The original contributions presented in the study are included in the article/supplementary material. Further inquiries can be directed to the corresponding author.
